# Effects of physical activity level on quality of life, stress, and dietary behavior in people living with HIV/AIDS: A pilot study

**DOI:** 10.1097/MD.0000000000033460

**Published:** 2022-04-07

**Authors:** Sung-Hoon Kim, Chang-Hwa Joo

**Affiliations:** a Department of Physical Education, Yonsei University, Seoul, South Korea; b Department of Sport Science, Kangwon National University, Chuncheon, South Korea; c Department of Smart Health Science and Technology Active Senior Customized Smart Healthcare Convergence Technology Professional Training Project Team, Kangwon National University, Chuncheon, South Korea.

**Keywords:** dietary behavior, HIV, physical activity, quality of life

## Abstract

The aim of this study was to analyze the daily physical activity and the relationship between the physical activity levels and psychological variables of Koreans with HIV. Twenty-two people with HIV participated in this study. The participants completed questionnaires, and we assessed the daily physical activity for 2 weeks. The daily physical activity level of most of the participants was low-intensity; high-intensity activities were only performed for approximately 1 minute. The participants had unhealthy dietary habits, such as eating 2 meals a day, eating irregularly, and skipping breakfast. Psychological well-being and environmental satisfaction were higher in the high-intensity group than in the medium- and low-intensity groups (*P* < .05). The overall stress level among the groups was statistically significantly different (*P* < .05), and the stress level was lower in the high-intensity group than in the low- and medium-intensity groups. Restraint eating was higher in the low-intensity group than in the medium- and high-intensity groups (*P* < .05). However, among the groups, the external eating variable was highest in the high-intensity group (*P* < .05). Daily physical activity during positively affects the physical and mental well-being of people living with HIV.

## 1. Introduction

The amount of physical activity in daily life, including exercise, is closely related to health improvement. Regular exercise reduces cardiovascular risk factors, improves fitness, and maintains health status.^[1]^ Additionally, exercise reduces weight and various risk factors related to diseases, such as hypertension in obesity, which cause various diseases.^[2,3]^ Furthermore, exercise lowers blood glucose levels by enhancing insulin sensitivity, even in people with diabetes.^[4]^ Notably, high volumes of moderate activity in daily life reduce disease risks and mortality in sedentary people.^[5]^ Even people with cardiovascular disease and cancer can gain substantial longevity benefits by increasing the amount of daily physical activity.^[6]^

Although the amount of daily physical activity is closely related to health, people with HIV tend to avoid social activities due to the stigmatization of the infection. The level of participation in physical activities in daily life may vary depending on the level of immunity of patients due to the HIV infection; fewer CD4 cells restrict many daily life activities, such as walking, eating, and driving.^[7]^ These restrictions and reduced physical activity, including exercise, may be related to fatigue in people with HIV. A previous study investigated the relationship between fatigue level and physical activity in people with HIV; a lower fatigue level was associated with a higher amount of daily physical activity.^[8]^ Additionally, the daily physical activity duration of people with HIV was 144 ± 31 minutes, and those who were more physically active had healthier lifestyles than that had by those who did not.^[9]^ Therefore, people with HIV need to increase their daily physical activity, such as walking upstairs rather than using an elevator, to improve their immune function and physical fitness.

Perceived quality of life (QoL) should be high for human beings to lead happy lives. QoL is associated with the amount of participation in daily physical activities. The limitation of these activities by chronic pain reduces QoL.^[10]^ Health-related QoL is higher in persons who participate in recommended levels of physical activities than in inactive and insufficiently active persons.^[11]^ Additionally, physical activity-related QoL may vary depending on the level of physical activity. People with high daily physical activity levels have a higher QoL than that had by those with low levels.^[12]^ A similar relationship between physical activity and QoL was observed in people with HIV; a study reported that more physical activity improves QoL in people with HIV.^[13]^

Nutrition combined with exercise is important for improving health. Proper eating habits, such as a balanced nutritional intake, can promote health and naturally increase immunity.^[14]^ However, the low-income class has a higher consumption rate of instant food and obesity than that had by the high-income class.^[15]^ Although optimal nutrient intake is paramount to people living with HIV, many are prone to malnutrition or obesity due to inadequate nutritional intake, a major cause of high mortality in people living with HIV.^[16]^ Consequently, education programs on physical activity and nutrition in daily life for people with HIV will greatly help improve their health and QoL. Nevertheless, it is necessary to identify the current level of physical activity and nutritional intake among people with HIV to organize this educational program. However, no studies have examined the level of physical activity and nutritional intake among Koreans with HIV. Therefore, it is necessary to research the lifestyle (physical activity and nutrition intake) and the relationship between the level of physical activity and psychological variables in South Koreans with HIV.

## 2. Materials and methods

### 2.1. Participants

Twenty-two people with HIV participated in this study and were recruited in collaboration with the Korea AIDS Prevention Association. Study-related procedures were explained to participants, and those who expressed willingness to participate completed a consent form. Ethical approval was granted by the Ethics Committee of Kangwon National University (KWNUIRB-2020-11-003-001).

### 2.2. Study procedures

The test consisted of 3 stages. Step 1: Filling out the questionnaire and measure body composition; Step 2: Wearing an activity monitoring device throughout daily life; and Step 3: Visiting the lab and returning the device.

After arriving at the laboratory, participants sat down and rested for at least 30 minutes. Next, they listened to an explanation of the procedure of the study and how to fill out the questionnaire and body composition will be analyzed (body composition device: Inbody 470; InBody Co. Ltd., Seoul, Korea). Next, the researcher explained the questions to participants who did not understand the questions while filling out the questionnaire. All questionnaires were completed under confidentiality. After completing the questionnaire, the participants were taught how to use the device to analyze the amount of daily activity, and each participant operated the assigned device. Participants who had difficulty operating the device were taught again. Lastly, the activity monitoring devices were distributed to participants, and they wore them on their waist belts for 2 weeks, except during sleeping and showering. During this period, problems with operating the device were resolved over the phone, and the device was replaced if unresolved. After 2 weeks, they visited the experiment site and returned the device.

### 2.3. Activity monitoring

The amount of daily activities during the 2 weeks was analyzed after connecting the device (Fitmeter; Fit.Life, Suwon, Korea) to the computer using a software (Fitmeter manager 2; Fit.Life). The variables for activity analysis were based on total kcal expenditure, kcal expenditure during activity, period of activity (low: > 3 metabolic equivalent of task [MET], moderate: 3–6 MET, and high activity: > 6 MET groups),^[17]^ and level of daily activity, classified into low (201–520 kcal), medium (521–770 kcal) and high (>770 kcal) intensities based on energy expenditure during activities.^[18]^

### 2.4. Questionnaires

Demographic characteristics included age, marital status, occupation, education, and income level. Dietary habits were modified and supplemented for the items on the food intake behavior used in a previous study to suit the participants.^[19]^ It comprised 16 items on the number, regularity, amount, duration, time, skipped meals, frequency of eating out, frequency and type of snacks, number of late-night snacks, and unbalanced eating.

We used the Dutch dietary behavior questionnaire^[20]^ translated into Korean with verified validity and reliability. Furthermore, the questionnaire consists of 33 questions: 10 about abstinent eating to assess the extent to which weight can be checked by controlling food intake; 13 on emotional eating to check the effects of negative emotional states, such as anger, fear, and anxiety on eating behavior, and smell and taste; and 10 on the degree to which external stimuli induce eating behavior. Moreover, Cronbach’s alpha values for the dietary behavior questionnaire were identified as α = 0.93 (restraint), α = 0.92 (emotional), and α = 0.99 (external eating).

Additionally, we used the stress questionnaire developed by the Centers for Disease Control in Korea for analysis of the National Health and Nutrition. It comprises 3 factors: exhaustion (α = 0.96), depression (α = 0.84), and anger (α = 0.90), with a total of 20 questions, and response are provided on a 5-point scale. Regarding the QoL, the Korean version of the WHO QoL-HIV Brief developed by the WHO in 2002 was used; it comprises 6 areas including physical satisfaction (α = 0.88), psychological well-being (α = 0.91), independent satisfaction (α = 0.87), social relationship satisfaction (α = 0.95), environmental satisfaction (α = 0.92), and spiritual satisfaction (α = 0.90) with a total of 31 questions.

### 2.5. Statistical analysis

The data were analyzed using SPSS (ver 23.0; SPSS ICC, Chicago, IL). Demographic variables and the amount of physical activity of people with HIV are expressed as mean values ± standard deviation (mean ± SD) through frequency analysis. To determine the validity and reliability of the questionnaire measurement tool, a group of experts comprising 3 professors (health science, sports psychology, and exercise physiology) assessed content validity, and Cronbach’s α coefficient was calculated to determine reliability. Before analyzing the differences in stress and QoL according to the amount of physical activity using one-way ANOVA, a homogeneity test was conducted using Levin’s assumption of equal variance. Lastly, the Scheffé test was performed when equal variance was assumed; otherwise, the Tamhane test was performed. The significance level was set at *P* ≤ .05.

## 3. Results

The demographic variables of the study participants are listed in Table 1. The participants’ ages were widely distributed from <30 to >60 years. Although 22.7% of the participants had been married, the majority (77.3%) were not. Those married did not maintain their married status due to spousal death, separation, or divorce. Moreover, high school graduates accounted for the largest number of participants, followed by college graduates, middle school, and elementary graduates. None of the participants had full-time jobs, and most were unemployed. Lastly, one participant was paid > $800 per month, and all others were paid < $800 per month.

**Table 1:** Individual demographic characteristics (n = 22).

	Information	N (%)
Age (yr)	Under 30s	4 (18.2)
	40s	5 (22.7)
	50s	9 (40.9)
	Over 60s	4 (18.2)
Marital status	Single	17 (77.3)
	Married	–
	Widowed	1 (4.5)
	Separation	1 (4.5)
	Divorced	3 (13.6)
Education	Elementary graduate	3 (13.6)
	Middle school graduate	4 (18.32)
	High school graduate	10 (45.5)
	College or higher	5 (22.7)
Occupation	Temporary employed	3 (13.6)
	Unemployed	19 (86.4)
Monthly household income	Recipient of basic living ($430)	11 (50.0)
	<$800	10 (45.5)
	≥$800	1 (4.5)

Most of the participants’ daily physical activity was low-intensity; high-intensity activities were performed for approximately 1 minute (Table 2). Although there was no statistical difference between weekdays and weekends in all variables related to physical activity, calorie expenditure during activity (*P* = .07) and amount of low-intensity activity (*P* = .06) tended to be higher on weekends than on weekdays. Regarding classifying participants by daily physical activity level, medium activity level (13 participants; height: 172.1 ± 6.7 cm, weight: 71.3 ± 11.4 kg, body mass index [BMI]: 23.9 ± 2.6 kg/m^2^) was the most common, followed by low- (7 participants; height: 173.6 ± 5.3 cm, weight: 77.4 ± 9.8 kg, BMI: 25.6 ± 1.9 kg/m^2^) and high-intensity (2 participants; height: 165.0 ± 11.0 cm, weight: 71.8 ± 26.2 kg, BMI: 25.4 ± 6.2 kg/m^2^) levels.

**Table 2:** Difference in activity profile between week and weekend (n = 22).

	Total kcal expenditure (kcal)	Activity kcal expenditure (kcal)	Low activity (min)	Moderate activity (min)	High activity (min)
Week	2345.9 ± 540.2	519.0 ± 210.2	71.8 ± 37.5	42.7 ± 27.9	0.8 ± 3.4
Weekend	2272.3 ± 473.0	563.1 ± 223.6	78.2 ± 43.3	44.6 ± 28.2	1.6 ± 5.4
Total	2334.1 ± 523.3	534.1 ± 209.4	73.6 ± 40.2	43.6 ± 26.6	1.1 ± 3.5

Furthermore, 36.3% of the participants regularly ate 3 meals a day (Table 3). Additionally, breakfast was the most frequent meal for participants who did not eat 3 meals in a day. Lack of appetite was the most common reason for not eating breakfast, followed by oversleeping and habitual reasons. Moreover, the frequency of overeating was the highest (1–2 times per week), and the most overate meal was dinner. Lastly, the most preferred snacks were fruit juice and bread or cake. Most participants responded that they ate a balanced diet, and 6 ate an unbalanced diet.

**Table 3:** Individual dietary habits (n = 22).

	Sub-variable	N (%)		Sub-variable	N (%)
Number of meals (d)	1	2 (9.1)	Number of overeating	3 times < (d)	1 (4.5)
	2	12 (54.5)		2 times (d)	2 (9.1)
	3	7 (31.8)		1 time (d)	2 (9.1)
	4	1 (4.5)		3–4 times (wk)	1 (4.5)
Meal time	Very irregular	6 (27.3)		1–2 times (wk)	7 (31.8)
	Irregular	8 (36.4)		No overeating	9 (40.9)
	Regular	4 (18.2)	Overeating meal	Breakfast	2 (9.1)
	Very regular	4 (18.2)		Lunch	5 (22.7)
Amount of meal	Very scarce	3 (13.8)		Dinner	15 (68.2)
	Little lack	5 (22.7)	Number of eating out	1 time (d)	2 (9.1)
	Moderate	8 (36.4)		3–4 times (wk)	1 (4.5)
	Slightly full	4 (18.2)		1–2 times (wk)	7 (31.8)
	Very full	2 (9.1)		No eating out	12 (54.5)
Time period of meal	>10 min	4 (18.2)	Number of snack	3 times < (d)	1 (4.5)
	10–20 min	13 (54.5)		2 times (d)	5 (22.7)
	<20 min	6 (27.3)		1 time (d)	3 (13.6)
Skip a meal	Breakfast	15 (68.2)		5–6 times (wk)	1 (4.5)
	Lunch	3 (13.6)		3–4 times (wk)	3 (13.6)
	Dinner	1 (4.5)		1–2 times (wk)	4 (18.2)
	No skip	3 (13.6)		No snack	5 (22.7)
Number of breakfast per week	Every day	6 (27.3)	Snack time	Between breakfast and lunch	4 (18.1)
	5–6 d	4 (18.2)		Between lunch and dinner	3 (13.6)
	3–4 d	2 (9.1)		After dinner	7 (31.8)
	1–2 d	6 (27.3)		Anytime	8 (35.4)
	No breakfast	4 (18.2)	Type of snack	Bread or cake	4 (18.2)
Reasons for skipping breakfast	No skipping	4 (18.2)		Snack food	2 (9.1)
	Over sleeping	3 (13.6)		Flour based food	3 (13.6)
	No appetite	8 (36.4)		Fast food	1 (4.5)
	Indigestion	1 (4.5)		Fruit juice	6 (27.3)
	No preparing breakfast	2 (9.1)		Milk products	3 (13.6)
	Lose weight	1 (4.5)		Tea	1 (4.5)
	Habitually	3 (13.6)		Ice cream	2 (9.1)
Amount of rice	More than a bowl	5 (22.7)	Number of late-night meal	Every day	2 (9.1)
	A bowl	10 (45.5)		3–4 times (wk)	2 (9.1)
	2/3 bowl	3 (13.6)		1–2 times (wk)	7 (31.8)
	1/2 bowl	2 (9.1)		1–3 times (mo)	4 (18.2)
	1/3 bowl	2 (9.1)		No late-night meal	7 (31.8)
			Un-balanced feeding	Yes	6 (27.3)
				No	16 (72.7)

The QoL according to the level of physical activity is presented in Table 4. Although there was no statistically significant difference in the overall value of QoL, the high-intensity level group was at the “good” level; however, the low- and medium-intensity groups were “poor,” below the average. Among the QoL sub-variables, no differences were observed between groups in physical, independent, social-relational, and spiritual satisfaction. However, psychological well-being and environmental satisfaction were higher in the high-intensity group than in the medium- and low-intensity groups (*P* < .05).

**Table 4:** Differences in QoL according to level of physical activity (n = 22).

	Low activity (n = 7; A)	Medium activity (n = 13; B)	High activity (n = 2; C)	*F* value	Post-hoc
Physical satisfaction	2.8 ± 0.6	2.9 ± 0.4	3.6 ± 0.2	2.859	–
Psychological well-being	2.9 ± 0.7	2.5 ± 0.3	3.8 ± 0.1	7.411*	A, B < C
Independent satisfaction	2.8 ± 0.7	2.9 ± 0.6	3.5 ± 0.4	1.143	–
Social relational satisfaction	2.3 ± 0.8	2.4 ± 0.6	3.4 ± 0.5	2.148	–
Environmental satisfaction	2.5 ± 0.5	2.6 ± 0.7	3.8 ± 0.1	4.212*	A, B < C
Spiritual satisfaction	2.4 ± 0.6	2.7 ± 0.8	3.6 ± 0.2	2.848	–
Total	2.1 ± 0.4	2.2 ± 1.0	3.5 ± 0.7	2.413	–

*F* value represents one-way ANOVA results.

QoL = quality of life.

* Significance level of < .05.

No statistical differences were observed between groups in the stress sub-variables of burnout, depression, and anger (Table 5). However, a difference was observed in the overall stress level between the groups (*P* < .05), and the stress level was lower in the high-intensity group than in the low- and medium-intensity groups.

**Table 5:** Differences in stress according to the level of physical activity (n = 22).

	Low activity (n = 7; A)	Medium activity (n = 13; B)	High activity (n = 2; C)	*F* value	Post-hoc
Burnout	3.5 ± 0.3	3.4 ± 0.4	2.8 ± 0.2	2.466	–
Depression	3.5 ± 0.3	3.4 ± 0.5	2.8 ± 0.2	2.265	–
Anger	3.5 ± 0.3	3.2 ± 0.4	3.0 ± 0.4	2.561	–
Total	3.5 ± 0.3	3.3 ± 0.3	2.9 ± 0.3	4.663*	A, B > C

*F* value represents one-way ANOVA results.

* Significance level of < .05.

Among the sub-variables of dietary behavior, differences were observed between groups in restraint and external eating (*P* < .05; Table 6). Additionally, restraint eating was higher in the low-intensity group than in the medium- and high-intensity groups (*P* < .05). However, among the groups, the external eating variable was the highest in the high-intensity group (*P* < .05), and emotional eating did not differ between groups.

**Table 6:** Differences in dietary behavior according to levels of physical activity (n = 22).

	Low activity (n = 7; A)	Medium activity (n = 13; B)	High activity (n = 2; C)	*F* value	Post-hoc
Restraint eating	3.8 ± 0.3	3.2 ± 0.7	2.8 ± 0.3	3.998*	A > B, C
Emotional eating	2.2 ± 0.8	2.4 ± 1.2	4.0 ± 0.1	2.376	–
External eating	2.6 ± 0.7	2.6 ± 0.5	3.9 ± 0.5	4.394*	A, B < C
Total	2.9 ± 0.4	2.7 ± 0.7	3.5 ± 0.3	1.342	–

*F* value represents one-way ANOVA results.

* Significance level of < .05.

## 4. Discussion

This study aimed to analyze the daily physical activity and the relationship between the level of physical activity and psychological variables of Koreans with HIV. Most of participants in the study performed low- and medium-intensity physical activities, and only 9% of the participants performed high-intensity physical activities. The participants’ physical activity intensity level was closely related to QoL and stress. Higher QoL, and lower stress were observed in the high-intensity physical activity group than in the other groups.

Many participants had never been married, and those married in the past could not continue the marriage due to the death of a spouse, separation, or divorce. It is difficult to maintain stability and opportunities for marriage due to the social stigmatization of people with HIV.^[21]^ Furthermore, approximately 70% of the participants had high-level education, such as high school, college, or post-graduate study. This level of education was higher than that of people living with HIV in other countries, many of whom do not even complete high school.^[22]^ This phenomenon might be due to a stable education system in Korea such as compulsory education up to middle school and people’s preference to get a high level of education. Notably, educational level affects income across generations of parents and children in Korea.^[23]^ However, in this study, the participants had a high level of education, but none had a stable job, and most were unemployed or part-time workers. Additionally, their income level was extremely lower than that of the general population due to this occupational situation. Moreover, half of them lived on government subsidies without income. Notably, in the Korean society, people with HIV are avoided with the prejudice that they can transmit the infection when they come in contact with others in the same space as in work life. Sadly, even those who have completed a high level of education have difficulty finding stable jobs and live on low wages due to this social stigmatization.

Daily energy expenditure and level of TNF-alpha are higher in persons with HIV than in the general population.^[24]^ However, in our study, the participants’ total daily energy expenditure was 2334 kcal, similar to that of the general population.^[25]^ This varying result can be attributed to the difference in the period for measuring energy expenditure. Energy expenditure was not measured during the sleeping time in the current study; however, it was measured for 24 hours in the previous study. Considering that energy is expended even during sleep through basal metabolism, the energy expenditure would increase if measured for 24 hours. Our result is consistent with the previous findings that hypermetabolism exists in people with HIV compared with the general population.^[24]^

It is well known that daily exercise and energy expenditure from physical activities is closely related to lifespan.^[18]^ The activity energy expenditure of the participants in this study was lower than that in a previous study (534 vs 621 kcal/d) targeting the general population.^[26]^ The difference in these results is difficult to directly compare because energy expenditure according to activity intensity level was not analyzed in the previous study; however, the previous study’s participants probably completed more activity. Conversely, high-intensity activities were performed for approximately 1 minute in the present study. HIV infection directly affects participation in physical activity, and environmental factors, such as educational opportunities and changes in economic wealth caused by infection, indirectly interfere with participation in physical activity.^[13]^ Notably, in the present study, only 2 participants expended >770 kcal for physical activity per day. Therefore, people with HIV need to increase the amount of daily physical activity to live a healthy life without physical limitations and to extend their lifespan.

Interestingly, although there was no statistically significant difference, a higher tendency of activity energy expenditure was observed on weekends than on weekdays in the present study. Additionally, the period of activity by intensities was longer on weekends. In a previous study that analyzed the general public’s activity energy expenditure during weekdays and weekends, the energy expenditure was similar between weekdays and weekends.^[27]^ Notably, the differences in activity energy expenditure between weekdays and weekends may be deeply related to the nature of participants’ occupations in the present study. As mentioned above, most participants were unemployed and did not need to move to work during the week and perform more physical activity by participating in hobbies during weekends.

QoL is an important consideration for the healthy life of people with HIV,^[28]^ and it goes beyond simple individual health status and considers social health. In other words, QoL is a multidimensional concept that encompasses physical, mental, and social well-being.^[29]^ However, physical symptoms of HIV infection (headache, pain, fatigue, nausea, diarrhea, and rash)^[30]^ and negative social interactions caused by cognitive, mental, and emotional disorders limit participation in social activities.^[31–33]^ Consequently, HIV infection inevitably lowers the QoL due to a lack of physical activity.^[34]^ The high-activity group was statistically higher than the other groups in psychological well-being and environmental satisfaction among the sub-variables of QoL in our study. These results suggest that individuals with a high level of physical activity increase their mental self-esteem by participating in various social and physical activities; hence, social interaction is carried out smoothly, and stress factors, such as anxiety and depression, are effectively managed. Additionally, it is understood that they try to evaluate their lives positively by having a supportive environment from family or people around them. Therefore, increasing leisure and physical activity participation, stress management, and social support are necessary to improve QoL.^[28]^

Perceived stress in people living with HIV is strongly related to state and trait anxiety, depression, HIV-related symptoms, sleep quality, and fatigue. Additionally, psychological factors have more influence on perceived stress than physiological factors, such as disease stage.^[35]^ The stress level was the lowest in the high-activity group in the current study. These results confirm previous studies that physical activity reduces symptoms of depression and control anxiety through changes in mood and cognitive function.^[36–38]^ Additionally, it positively affects the physical and mental health of people with HIV.^[32,34,39–41]^ From a physiological perspective, regular physical activity stimulates the parasympathetic nervous system activity, reduces due to anxiety, and ultimately controls anxiety symptoms.^[42]^ Overall, the regular physical activity of people with HIV can prevent anxiety and depression and helps manage stress.

This study has some limitations. The main limitation was the small number of study participants and the wide age distribution from <30 to >60 years. Therefore, there were limitations in revealing the characteristics of a specific age group. Furthermore, the statistical reliability of this study is low due to the small number of participants and 3 groups according to the level of physical activity. Additionally, since the participants in the study were recruited from a specific region (2 cities), the results do not represent the situation of people with HIV living in the entire South Korea. Although we conducted this study and recruited the participants in collaboration with the local AIDS Prevention Association, it was difficult to recruit them because they did not want to expose their infection. Therefore, future studies researching basic information, such as lifestyle, and analyzing the relationship between the level of physical activity and QoL should use many people with HIV in various regions.

The study participants had unhealthy dietary habits, such as eating 2 meals a day, eating irregularly, and skipping breakfast. Notably, a family’s income level can affect eating habits, such as skipping breakfast.^[43,44]^ Therefore, participants’ eating habits may be related to their occupational and economic circumstances. All participants did not have full-time jobs, and their income level was lower than that of the general population. Furthermore, bad dietary habits due to insufficient economic circumstances are maintained by skipping breakfast in the morning as there is no need for activities, such as going to work. Dietary behavior is evaluated as an essential factor in explaining obesity due to excessive nutritional intake, such as overeating and binge eating.^[45]^ Physical activity is essential for weight management; therefore, it is important to understand dietary behavior according to the physical activity level of persons with HIV. The low-activity group had higher restrained eating than that had by the other groups. A high restrained eating score prevents weight gain and maintains a BMI score.^[46–48]^ Considering these results, it is deduced that the low-activity group managed weight and health through restrained eating rather than physical activity. Notably, there were no statistically significant differences in body weight and BMI between the groups in this study.

Furthermore, a higher external eating behavior score was found in the high-level active group than in the others. Although previous studies on the relationship between eating behavior and body weight have shown that emotional and external eating behaviors can lead to overeating and weight gain or obesity,^[49–52]^ the high-activity group may prefer eating by external stimuli, such as delicious food, regardless of the internal state (hunger or satiety) rather than worrying about obesity due to increased physical activity. Few studies have been conducted on dietary behavior in persons with HIV. Therefore, the findings of this study can provide basic data for future research on dietary habits and behaviors for the health management of people with HIV.

## 5. Conclusion

The energy expenditure level of Koreans infected with HIV during daily life did not reach an ideal level, and the duration of high-intensity activity was remarkably low. Additionally, poor dietary habits, such as skipping breakfast, were observed. Furthermore, people with a high level of physical activity had a higher level of QoL and managed stress more effectively than that observed in the low-level activity group. Overall, physical activity during daily life positively affects the physical and mental well-being of people living with HIV. Therefore, the government needs to develop and provide physical activity programs enabling people with HIV to participate in physical activities voluntarily without financial burden, and continuous support from surrounding people should be provided.

## Author contributions

**Conceptualization:** Chang-Hwa Joo.

**Data curation:** Sung-Hoon Kim.

**Funding acquisition:** Chang-Hwa Joo.

**Investigation:** Sung-Hoon Kim, Chang-Hwa Joo.

**Methodology:** Sung-Hoon Kim.

**Writing – original draft:** Sung-Hoon Kim, Chang-Hwa Joo.

**Writing – review & editing:** Chang-Hwa Joo.
